# Optimum Temperatures for Net Primary Productivity of Three Tropical Seagrass Species

**DOI:** 10.3389/fpls.2017.01446

**Published:** 2017-08-23

**Authors:** Catherine J. Collier, Yan X. Ow, Lucas Langlois, Sven Uthicke, Charlotte L. Johansson, Katherine R. O'Brien, Victoria Hrebien, Matthew P. Adams

**Affiliations:** ^1^Centre for Tropical Water and Aquatic Ecosystem Research, James Cook University Cairns Cairns, QLD, Australia; ^2^College of Marine and Environmental Sciences, James Cook University Townsville Townsville, QLD, Australia; ^3^Australian Institute of Marine Science Townsville, QLD, Australia; ^4^School of Chemical Engineering, The University of Queensland Brisbane, QLD, Australia

**Keywords:** net primary productivity, thermal stress, sea temperature, climate change, tropical seagrass, *Cymodocea serrulata*, *Halodule uninervis*, *Zostera muelleri*

## Abstract

Rising sea water temperature will play a significant role in responses of the world's seagrass meadows to climate change. In this study, we investigated seasonal and latitudinal variation (spanning more than 1,500 km) in seagrass productivity, and the optimum temperatures at which maximum photosynthesis and net productivity (for the leaf and the whole plant) occurs, for three seagrass species (*Cymodocea serrulata, Halodule uninervis*, and *Zostera muelleri*). To obtain whole plant net production, photosynthesis, and respiration rates of leaves and the root/rhizome complex were measured using oxygen-sensitive optodes in closed incubation chambers at temperatures ranging from 15 to 43°C. The temperature-dependence of photosynthesis and respiration was fitted to empirical models to obtain maximum metabolic rates and thermal optima. The thermal optimum (*T*_opt_) for gross photosynthesis of *Z. muelleri*, which is more commonly distributed in sub-tropical to temperate regions, was 31°C. The *T*_opt_ for photosynthesis of the tropical species, *H. uninervis* and *C. serrulata*, was considerably higher (35°C on average). This suggests that seagrass species are adapted to water temperature within their distributional range; however, when comparing among latitudes and seasons, thermal optima within a species showed limited acclimation to ambient water temperature (*T*_opt_ varied by 1°C in *C. serrulata* and 2°C in *H. uninervis*, and the variation did not follow changes in ambient water temperature). The *T*_opt_ for gross photosynthesis were higher than *T*_opt_ calculated from plant net productivity, which includes above- and below-ground respiration for *Z. muelleri* (24°C) and *H. uninervis (*33°C), but remained unchanged at 35°C in *C. serrulata*. Both estimated plant net productivity and *T*_opt_ are sensitive to the proportion of below-ground biomass, highlighting the need for consideration of below- to above-ground biomass ratios when applying thermal optima to other meadows. The thermal optimum for plant net productivity was lower than ambient summer water temperature in *Z. muelleri*, indicating likely contemporary heat stress. In contrast, thermal optima of *H. uninervis* and *C. serrulata* exceeded ambient water temperature. This study found limited capacity to acclimate: thus the thermal optima can forewarn of both the present and future vulnerability to ocean warming during periods of elevated water temperature.

## Introduction

Rising sea surface temperature caused by global climate change threatens coastal and marine communities around the world. Tropical regions are particularly vulnerable as thermal anomalies cause coral bleaching and mortality of coral reefs, which, together with decreased pH (“acidification”) of the oceans will continue to modify tropical marine habitats and their ecosystem services (Doney et al., 2011; Hoegh-Guldberg et al., 2014). Although many seagrass species are able to tolerate higher temperatures than coral reef habitats are able to tolerate (Pörtner et al., 2014), there are seagrass meadows living close to their thermal limits that are at risk from rising temperatures (Massa et al., 2009; Collier et al., 2011; Pedersen et al., 2016; Repolho et al., 2017). Increases in annual temperature of less than 3°C have induced seagrass mortality in both temperate (Marbá and Duarte, 2010) and subtropical locations (Thomson et al., 2015). Similarly, multi-decadal variation in climate-driven changes in temperature and rainfall were associated with changes in seagrass meadow area and abundance in far northern Australia (Rasheed and Unsworth, 2011). These emerging climate pressures exacerbate the “global crisis” for seagrass ecosystems caused by localized stressors such as water quality (Orth et al., 2006; Waycott et al., 2009) and are predicted to induce functional extinction of some seagrass meadows within the next few decades (Jorda et al., 2013). Temperature-induced seagrass loss may compromise the socio-ecological functions of seagrass meadows: habitat for fisheries species; food for herbivores including dugong, manatees and turtle; shoreline protection; a globally significant carbon stock; and, removal of potential pathogens (Heck et al., 2008; Marsh et al., 2011; Pergent et al., 2014; Dewsbury et al., 2016; Lamb et al., 2017).

Seagrasses as a functional group inhabit broad temperature ranges from 0 to 45°C, and particular species can tolerate ambient water temperature that varies by more than 20°C annually (Lee et al., 2007). They are tolerant of these broad temperature ranges due to protection and repair mechanisms that enable temperature-specific optimization of vital metabolic functions including photosynthesis; however, these protective mechanisms cease to afford protection under heat stress (Reusch et al., 2008; Bita and Gerats, 2013; Yamori et al., 2014). Therefore, photosynthetic rates increase with temperature prior to reaching a maximum rate at the thermal optimum (Marsh et al., 1986; Lee et al., 2007). At elevated temperatures, stress responses, including production of stress proteins and mitochondria, increase respiratory rates (Bita and Gerats, 2013; Koutalianou et al., 2015). In combination with reduced photosynthetic carbon fixation at temperatures greater than the optima these respiratory heat stress responses result in declining net productivity under extreme temperatures (Marsh et al., 1986; Staehr and Borum, 2011; Pedersen et al., 2016). Net productivity reflects the assimilation of resources that become available for growth and biomass production. Defining the temperature-dependency of net productivity is important for the development of growth models (Baird et al., 2016), for setting environmental targets (Eakin et al., 2009), and to identify habitats at risk from thermal stress (Anthony et al., 2009).

The optimum temperature for seagrass growth affects seasonal growth dynamics and species-specific distributional ranges (Campbell et al., 2006; Lee et al., 2007; Marbá and Duarte, 2010; Collier et al., 2011). In a review of thermal optima, subtropical to tropical species reached maximum rates of photosynthesis at 23 to 32°C (Lee et al., 2007). However, thermal optima for some tropical species are even higher, for example, net productivity of *Halodule uninervis* increased between 30 and 33°C after 4 weeks (Collier et al., 2011) while quantum efficiency and shoot density of *Halodule wrightii* was unaffected at 34–35°C relative to cooler temperatures after 38 days (Koch et al., 2007).

Seagrasses can demonstrate phenotypic plasticity (McDonald et al., 2016), which could affect optimum temperatures for net productivity. In the temperate species *Zostera marina*, thermal optima were found to adjust over seasonal temperatures (Staehr and Borum, 2011), which suggests that seagrass may have the capacity to thermally acclimate to longer-term ocean warming induced by climate change. This conjecture is also supported by observations of complete thermal acclimation of seagrass productivity to 10 and 20°C in the laboratory (Zimmerman et al., 1989).

Several previous studies (e.g., Kerr and Strother, 1985; Masini and Manning, 1997; Massa et al., 2009; York et al., 2013; Kaldy, 2014) provide temperature thresholds in the format of a “temperature range,” but have been unable to precisely define thermal optima due to limitations of study design. To overcome this, Pedersen et al. (2016) and Staehr and Borum (2011) used curve-fitting to calculate thermal optima from net productivity measured over a broad temperature range. Thermal optima of leaf net productivity for tropical species were 32.8 ± 0.6 and 33.3 ± 0.8°C for the tropical *Thalassia hemprichii and Enhalus acroides*, respectively (Pedersen et al., 2016). Carefully considered model selection can ensure that the best estimates of thermal optima for seagrass productivity are obtained (Adams et al., 2017).

Thermal optima of seagrass productivity based only on leaf-scale processes may not represent thermal optima of the whole plant, since seagrasses have non-photosynthetic compartments (rhizome and roots) below the sediment that may account for the majority of their total biomass (Duarte and Chiscano, 1999). Below-ground biomass of seagrass therefore must be considered when calculating energetic budgets for the whole plant (Fourqurean and Zieman, 1991). Below-ground components are used for lateral expansion, clonal integration (Prado et al., 2008), and storage of carbon as carbohydrates (Burke et al., 1996; Touchette and Burkholder, 2000). Respiratory carbon loss from metabolic activity in leaves and rhizomes can exceed photosynthesis at high temperatures (Masini and Manning, 1995; Collier et al., 2011), and hence, non-photosynthetic components can place a respiratory burden on plants (Fourqurean and Zieman, 1991). Thus, below-ground non-photosynthetic tissue can have a large influence on net productivity and could also affect thermal optima. However, below-ground tissues are neglected in many studies investigating temperature dependent metabolism and thermal optima in seagrasses.

In this study, we identified the net productivity and thermal optima of three seagrass species: *C*. *serrulata, H. uninervis*, and *Z. muelleri*. These species are widely distributed and ecologically important but the thermal optima for their productivity were not previously known. We also investigated how the net productivity and thermal optima are affected by ambient water temperature. To accomplish this, photosynthesis and respiration of above-ground and below-ground tissues of seagrass were measured using oxygen sensitive optodes at sites separated by more than ~1,500 km along a latitudinal gradient and during austral summer and winter. We hypothesized that (1) thermal optima would be variable among species, (2) thermal optima would acclimate to ambient water temperature, both across seasons and latitude, and (3) accounting for seagrass respiration would reduce estimates of their thermal optima. Environmental managers can use the thermal optima and net productivity values reported here to identify the vulnerability of these seagrasses to ocean warming.

## Methods

### Study sites

**Figure 1:**
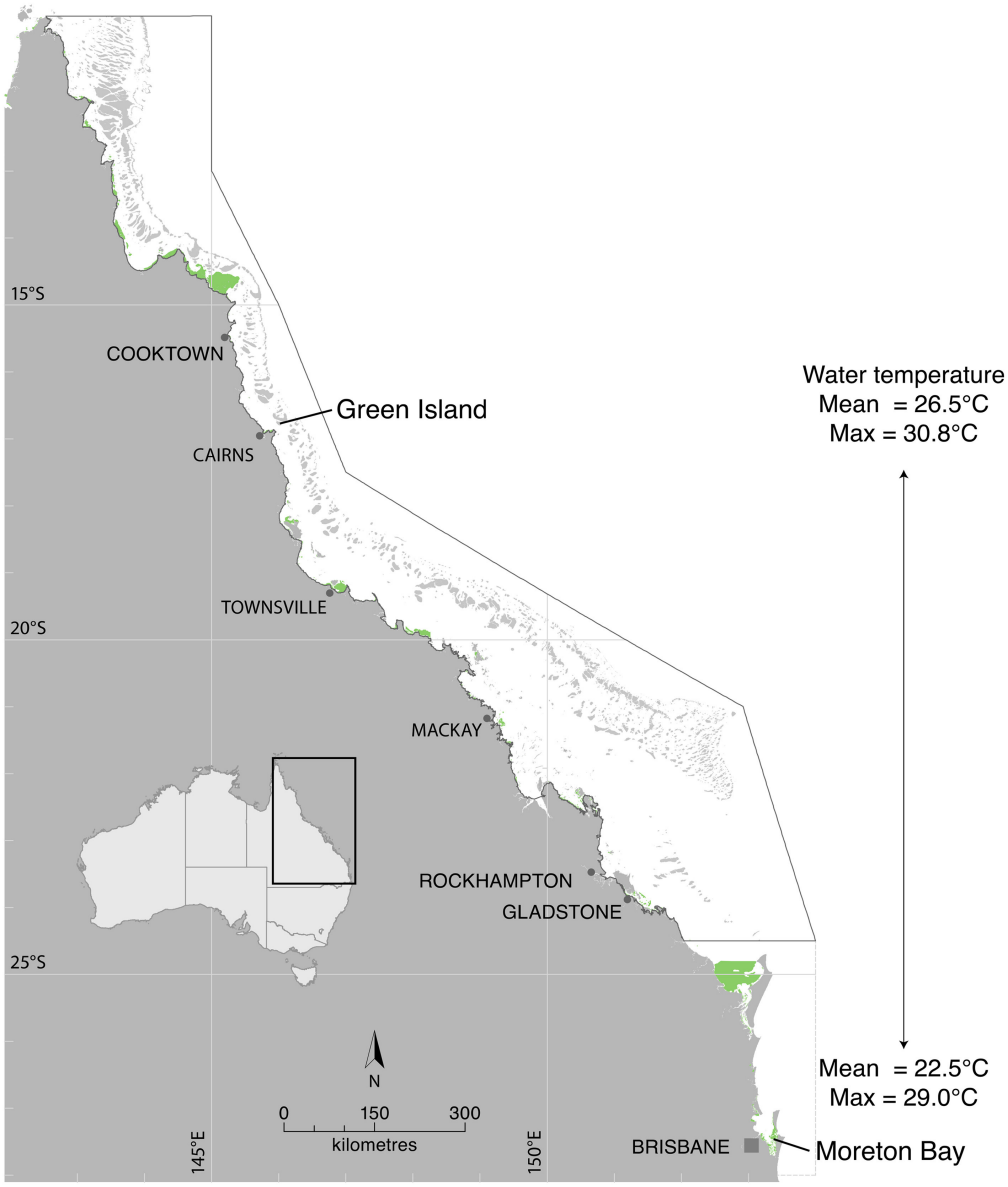
Photosynthesis and respiration were measured in the laboratory from seagrasses collected from Green Island in the northern Great Barrier Reef and from Moreton Bay. Seagrass distribution is shown in green from McKenzie et al. (2014) and Roelfsema et al. (2014).

The study was undertaken under conditions of varying ambient water temperature to include both latitudinal and seasonal comparisons. Collection sites for laboratory measurements of seagrass productivity were Green Island, a tropical site in the northern Great Barrier Reef (16° 45.29′S 145° 58.38′E), and a sub-tropical site ~1,500 km (latitudinal distance ~10.5°) to the south in Moreton Bay (27° 29.53′S 153° 24.09′E) hereafter referred to as “latitude” treatments (Figure 1). Temperature-production curves were measured in summer (January 2015 at Green Island and February/March 2015 at Moreton Bay) and winter (June 2015 at Moreton Bay), for comparison between seasons. Water temperature at the study sites was recorded at the canopy level with autonomous iBTag (AlphaMach, Canada) submersible temperature loggers. Mean water temperature at Green Island from 2008 to 2015 was 26.5°C and ranged from 21.2 to 30.8°C (based on continuous logging within the meadow, McKenzie et al., 2016). At Green Island, mean temperature in the 5 days preceding measurement of the temperature-production curves was 28.8°C in summer. Mean water temperature in Moreton Bay at Dunwich jetty adjacent to the study site from 2005 to 2014 was 22.5°C, ranging from 15.3 to 29.0°C (based on monthly spot measures at 0.2 m depth at site 502, Ecosystem Health Monitoring Program; http://hlw.org.au/report-card/monitoring-program). Mean temperature for 5 days preceding the measurements at nearby shallow seagrass meadows (Wanga-Wallen Banks, ~5 km away and at the same depth to the study site), was 27.2°C in summer and 21.0°C in winter.

### Photosynthesis and respiration temperature-production curves

Temperature-production curves, defined here as photosynthesis, net productivity or respiration at multiple different temperatures, were measured for *Cymodocea serrulata, H. uninervis*, and *Zostera muelleri*. Two of the species, *C. serrulata* and *H. uninervis*, occurred at both sites and in both seasons (Figure S1): *H*. *uninervis* was the most abundant species (percentage of total biomass ranged from 44 to 68% at both sites), followed by *C. serrulata* (10 to 39%), while *Z. muelleri* only occurred in Moreton Bay (13 to 15%). Whole shoots for measurement of temperature-production curves were collected from the center of the meadow patches less than 12 h prior to the incubation and held in re-circulation tanks at ambient water temperature fitted with gas bubblers.

Seagrass photosynthesis and respiration were measured in small incubation chambers using optical oxygen sensors (“optodes” PreSens, Sensor spots-Pst3) and two PreSens Oxy 4 four-channel oxygen meters (Presens, Germany). Two arrays of four chambers were run at each time. Each optode was calibrated following Collier et al. (2011) prior to initial measurements. Small transparent acrylic chambers (70 ml) were set in an array of four (i.e., four separate chambers allowing four parallel measures) and incubated at treatment water temperature using a flow-through water jacket system connected to a water bath (Lauda, Ecoline RE 106). Each chamber was stirred with a magnetic stirrer bar. A blank chamber was included in each array of four chambers to test for blank (i.e., water) respiration or production.

Whole shoots, including all leaves (ranging from 2 to 4 leaves depending on species), were used in incubations with the tips of leaves trimmed if, when fitted to the chamber, they would not maintain a vertical orientation. The leaves were held upright in the chamber by inserting the leaf through a small hole in the base of the chamber to mimic natural orientation. Oxygen consumption (dark respiration) was measured in the dark and photosynthetic rates were then measured on the same leaves at the same temperature in the light. The chambers were illuminated using white LED lamps at a saturating light level of 400 μmol m^−2^ s^−1^ (Ow et al., 2016) using a photosynthetically active radiation (PAR) probe (MQ-200, Apogee Instruments), which was calibrated against a manufacturer-calibrated 2π light sensor (LiCor™). This was repeated at increasing temperatures (7 steps) ranging from 15 to 43°C in winter and 17 to 43°C in summer. The water bath and temperature loggers were calibrated against a precision NATA certified mercury thermometer. A minimum of 40 min was allowed after changing the temperature of the water bath to enable the temperature of the incubation chambers to reach target temperature. Once target temperature was reached, the dark respiration of the leaf was measured followed by the photosynthetic rate. After that, the temperature of the bath was increased again. Each dark and light step lasted 20–40 min each with the specific time depending on when oxygen production or consumption had stabilized (i.e., oxygen concentration was linearly increasing or decreasing). Seawater within the chambers was refreshed prior to the last two temperature steps. Previous tests of the water discarded from the incubation chambers showed very small (<0.05 pH units) changes in chamber pH over the incubation period when using this water changing regime (Collier unpubl).

Oxygen concentration was logged every 15 s, and respective respiration and production rates were calculated by fitting a linear regression to the data. Oxygen flux in the blanks were used to offset the measured photosynthetic and respiration rates in chambers with leaves. After the incubation, leaves were rinsed in freshwater and dried for 48 h at 60°C to measure leaf dry weight, so that the measured photosynthesis and respiration rates could be normalized to leaf biomass.

Respiration rate in the rhizome and root complex of the same shoot (in lengths of ~5–10 cm) was measured subsequent to leaf measurements. The rhizome was secured in the chamber and dark respiration was measured following the same procedure as described for leaves; however, below-ground respiration was measured at four temperatures evenly distributed across the same temperature range.

### Biomass and growth

In order to estimate productivity at the plant scale based on seagrass leaf photosynthesis, leaf respiration and below-ground respiration, above-ground and below-ground biomass was measured at both sites within both seasons. At Green Island, biomass samples for *C. serrulata* were collected at a site 500 m away from the original site due to exceptionally low biomass of that species at the original site. A 20 cm diameter stainless steel corer was pushed into the sediment to a depth of 20 cm, and sediment, below-ground tissues and leaves were removed. The sediment was gently shaken in the water, the seagrass biomass was placed in a bag, and the samples chilled (4°C) or frozen (−16°C) prior to further processing within 3 days of collection. The sample was cleaned in freshwater, and epiphytes were removed by gentle scraping with a razor, before the material was sorted into species. Samples from each species were further separated into leaves (hereafter referred to as “above-ground”), below-ground (hereafter referred to as “below-ground”), and browned sheath that did not have leaves attached. The sheath was discarded. The samples were then dried at 60°C for 48 h, cooled in an airtight container and weighed.

### Mathematical methods

#### Fitting equations to the temperature-dependence of photosynthesis and respiration

**Figure 2:**
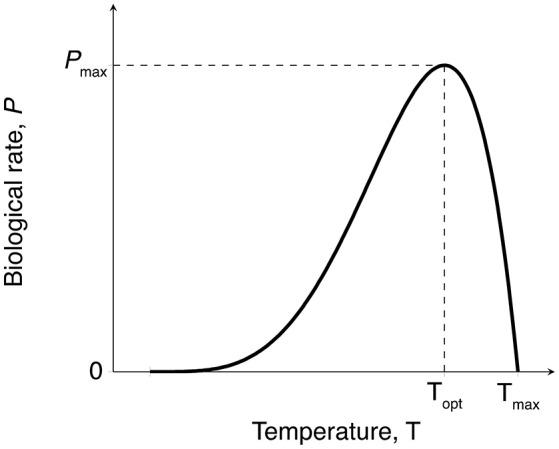
Expected temperature-dependence of gross photosynthesis and respiration rates for seagrass. Biological rates with this temperature-dependence can be fitted to the empirical model shown in Equation (1) to obtain maximum metabolic rates (*P*_max_), thermal optima (*T*_opt_), and maximum temperature (*T*_max_).

Both photosynthesis and respiration rates were expected to increase gradually with temperature up to an optimum (*T*_opt_), followed by a rapid decline of these rates at higher temperatures (Marsh et al., 1986; O'Sullivan et al., 2013) (Figure 2). We fitted the Yan and Hunt model (Yan and Hunt, 1999) to our data for photosynthesis vs. temperature and respiration vs. temperature, for seagrass above-ground and below-ground tissues, as this model has previously been fitted to data for both macrophyte growth rate (van der Heide et al., 2006) and photosynthesis (Adams et al., 2017),

(1) $$\begin{array}{r} P\left(T\right)=P_{\text{max}}\left(\frac{T_{\text{max}}-T}{T_{\text{max}}-T_{\text{opt}}}\right){\left(\frac{T}{T_{\text{opt}}}\right)}^{T_{\text{opt}}/\left(T_{\text{max}}-T_{\text{opt}}\right)} \end{array}$$

In this equation, *P*(*T*) is the biological rate *P* at temperature *T* (°C), and can represent either photosynthesis or respiration (mg C g^−1^ DW h^−1^), *P*_max_ is the maximum rate (mg C g^−1^ DW h^−1^) which occurs at the optimum temperature *T*_opt_ (°C), and *T*_max_ (°C) is the temperature greater than the optimum at which the biological rate drops to zero. For simplicity, we hereafter refer to the Yan and Hunt model as the productivity-temperature (PT) model.

Equation (1) was fitted to seagrass gross photosynthesis (*P*_gross(AG)_), leaf respiration (*R*_AG_), and below-ground respiration (*R*_BG_) against temperature using non-linear regression (MATLAB Statistics and Machine Learning Toolbox R2015b), for the three species and the latitude and season combinations. For each combination, the parameters obtained from this model fitting were only kept if the optimum temperature *T*_opt_ predicted by the PT model fell within the temperature range of the experiment (i.e., <43°C). We further rejected any model predictions of maximum temperature where the uncertainty bounds (±SE) in the thermal optimum overlapped with the uncertainty bounds in maximum temperature (±SE), as the predicted value of *T*_max_ in these cases possessed unsatisfactorily high uncertainty.

Below the optimum, the temperature dependence of biological rates can be characterized by the parameter Q_10_, which is the factor increase in biological rate due to a temperature increase of 10°C (Valiela, 1995). To estimate Q_10_ values, a second model was fitted to the temperature-dependence of seagrass gross photosynthesis (*P*_gross(AG)_), leaf respiration (*R*_*AG*_), and below-ground respiration (*R*_*BG*_) for each of the three species and latitude and season combinations. This was done only for data collected at temperatures (*T*) that fell below the value of *T*_opt_ predicted by the PT model fitted to the data. The second model was an exponential function of the form

(2) $$\begin{array}{r} P\left(T\right)=P_{0}Q_{10}^{\left(T-T_{0}\right)/10} \end{array}$$

where *P*_0_ (mg C g^−1^ DW h^−1^) is the biological rate at some reference temperature *T*_0_ (°C). We set *T*_0_ = 20°C, following the convention of Baird et al. (2016).

#### Estimating net productivity of the leaves, and total net productivity

All measures of seagrass productivity (including whole plant productivity) are reported in units of mg C g^−1^ DW h^−1^. Net seagrass leaf productivity *P*_net(AG)_ and net seagrass plant productivity *P*_net(AG+BG)_ can be estimated from gross photosynthesis *P*_gross(AG)_, leaf respiration *R*_G_, below-ground respiration *R*_BG_, and below-ground to above-ground biomass ratio BG/AG according to

(3) $$\begin{array}{r} P_{\text{net}\left(\text{AG}\right)}=\;P_{\text{gross}\left(\text{AG}\right)}-\;R_{\text{AG}} \end{array}$$

(4) $$\begin{array}{r} P_{\text{net}\left(\text{AG}+\text{BG}\right)}=\;P_{\text{gross}\left(\text{AG}\right)}-\;R_{\text{AG}}-\;\frac{BG}{AG}\;\times \;R_{\text{BG}} \end{array}$$

The temperature-dependence of *P*_net(AG)_ and *P*_net(AG+BG)_ can therefore be estimated if the temperature-dependence of the four terms on the right side of Equations (3) and (4) is known. We have already fitted two different models (PT and exponential, Equations 1 and 2) to the temperature dependence of the terms *P*_gross(AG)_, *R*_*AG*_, and *R*_*BG*_. For calculation of *P*_net(AG)_ and *P*_net(AG+BG)_, we used the PT model (Equation 1) fitted to *P*_gross(AG)_, *R*_AG_, and/or *R*_*BG*_ where *T*_opt_ < 43°C was predicted, and the exponential model (Equation 2) fitted to *P*_gross(AG)_, *R*_AG_, and/or R_BG_ where *T*_opt_ ≥ 43°C was predicted. For the remaining term on the right side of Equation (4), as the dependence of BG/AG ratio on temperature has not been documented, we calculated the temperature-dependence of *P*_net(AG+BG)_ for BG/AG ratio values that fall within the range of BG/AG ratios observed for the corresponding latitude, season, and species. Thus, calculations of *P*_net(AG+BG)_ using Equation (4) were used to predict how the below-ground to above-ground biomass ratio affects both the maximum net productivity of the plant and the optimum temperature at which this maximum net plant productivity occurs. Uncertainties in the maximum net productivity and associated optimum temperature were calculated from the standard error (SE) of the parameters fitted via non-linear regression to Equations (1) and (2), using standard formulae for propagation of uncertainty (Ku, 1966).

## Results

### Temperature dependence of gross photosynthesis and respiration

**Figure 3:**
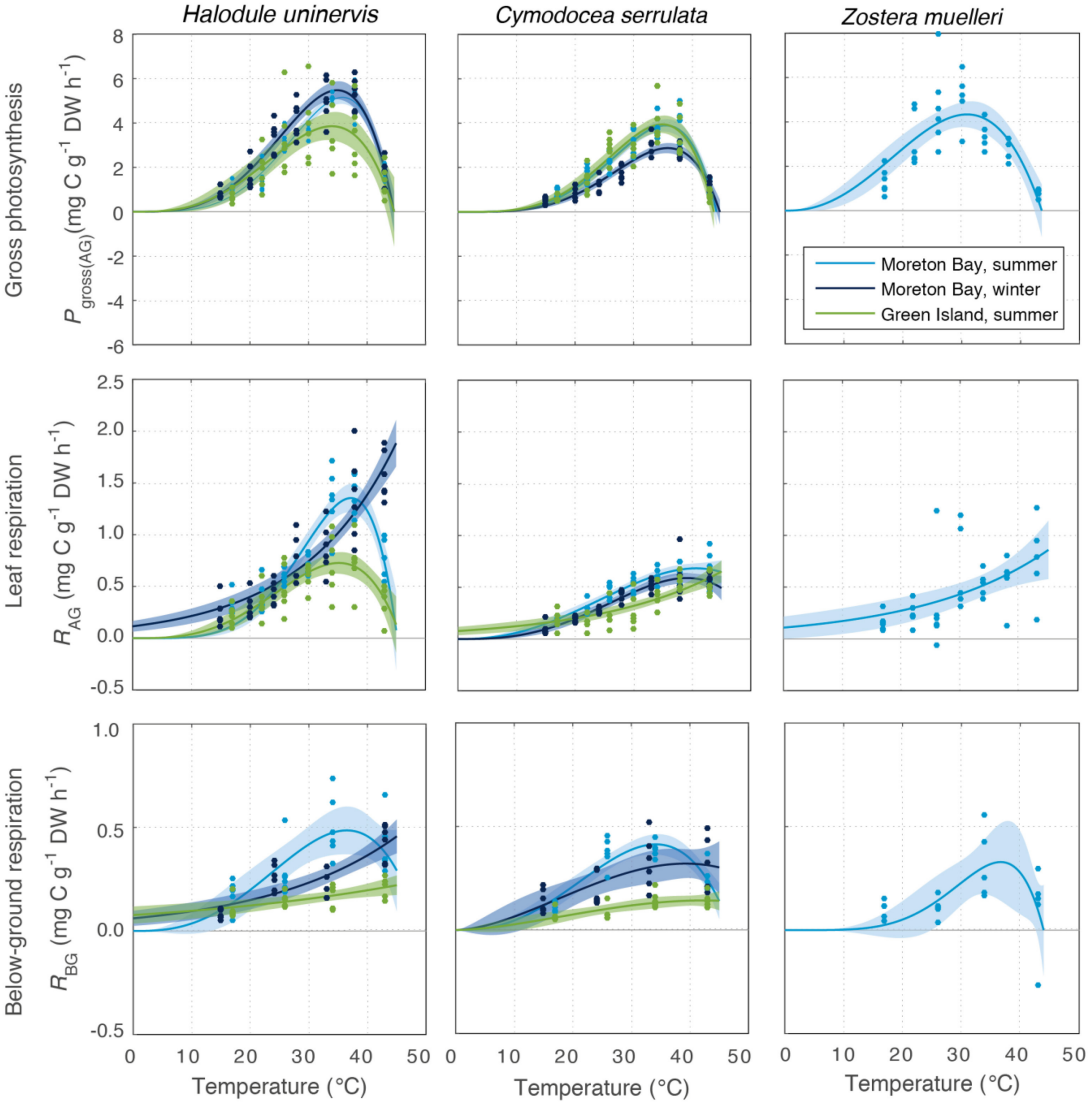
Gross photosynthesis (*P*_gross(AG)_, **Top**), and respiration in above-ground tissues (*R*_AG_) **(Middle)** and below-ground tissue (*R*_BG_) **(Bottom)** at water temperatures ranging from 15 to 43°C, for the seagrass species *H. uninervis, C. serrulata*, and *Z. muelleri*. These were measured at Green Island in summer and Moreton Bay in summer and winter. Data points shown for photosynthesis and net respiration (*n* = 6). Modeled fits for photosynthesis, and above-ground respiration and below-ground respiration, are shown by colored lines, and associated shaded error bounds indicate 95% CI in the model fits. Note the different scales on the y axes.

All species photosynthesized (i.e., had gross oxygen evolution rates > 0) at all temperatures tested (15–43°C, Figure 3). Gross photosynthesis *P*_gross(AG)_ followed a temperature-dependent relationship that can be described by the PT model (discussed in Adams et al., 2017). *P*_gross(AG)_ of seagrass leaves was predicted by the fitted PT model to be greater than zero for temperatures of up to 44–45°C in all species and season-latitude combinations (*T*_max_ in Table 1). At temperatures below the thermal optima, there was a gradual rise in *P*_gross(AG)_ with increasing temperature; this rise was characterized by the parameter Q_10_, the rate of increase per 10°C. The Q_10_ of *P*_gross(AG)_ ranged from 2.1 to 2.7 and was not substantially different between latitudes or seasons or among species (Figure S1.2, Table S1.1).

**Table 1:** Thermal optima, maximum productivity, and thermal maxima (±SD) found for photosynthesis and respiration of tropical seagrass.

	**Location, season**	**Productivity measure**	**Maximum rate, at *T_opt_* (mg C g^−1^ DW h^−1^)**	***T_opt_* (°C)**	***T*_max_ (°C)**
*H. uninervis*	Moreton Bay, Summer	*P*_gross(AG)_	5.2 ± 0.1	35.8 ± 0.2	44.4 ± 0.2
		*R*_AG_	1.4 ± 0.1	37.3 ± 0.4	45.2 ± 0.5
		*R*_BG_	0.5 ± 0.1	36.5 ± 1.2	49.2 ± 3.1
	Moreton Bay, Winter	*P*_gross(AG)_	5.5 ± 0.2	34.9 ± 0.3	44.6 ± 0.3
		*R*_AG_	1.6 ± 0.2^a^	>43	–
		*R*_BG_	0.4 ± 0.1^a^	>43	–
	Green Island, Summer	*P*_gross(AG)_	3.9 ± 0.3	34.0 ± 0.9	44.6 ± 0.9
		*R*_AG_	0.7 ± 0.1	35.2 ± 0.7	45.9 ± 1.0
		*R*_BG_	0.2 ± 0.1^a^	>43	–
*C. serrulata*	Moreton Bay, Summer	*P*_gross(AG)_	3.9 ± 0.1	35.4 ± 0.3	44.2 ± 0.3
		*R*_AG_	0.7 ± 0.0	40.7 ± 2.0	57.1 ± 4.5
		*R*_BG_	0.4 ± 0.0	34.2 ± 0.7	47.1 ± 1.0
	Moreton Bay, Winter	*P*_*gross*(*AG*)_	2.9 ± 0.1	35.8 ± 0.3	44.7 ± 0.3
		*R*_AG_	0.6 ± 0.0	39.1 ± 1.1	52.2 ± 2.6
		*R*_BG_	0.3 ± 0.0	39.3 ± 6.2	–^b^
	Green Island, Summer	*P*_*gross*(*AG*)_	4.0 ± 0.2	34.9 ± 0.5	43.7 ± 0.3
		*R*_AG_	0.5 ± 0.1*^a^*	>43	–
		*R*_BG_	0.1 ± 0.0	42.2 ± 9.7	–^b^
*Z. muelleri*	Moreton Bay, Summer	*P*_gross(AG)_	4.3 ± 0.3	30.9 ± 1.0	43.6 ± 0.7
		*R*_AG_	0.8 ± 0.4^a^	>43	–
		*R*_BG_	0.3 ± 0.1	36.7 ± 1.7	44.0 ± 0.9

a *If the model predicts T_opt_ > 43°C, the maximum rate is instead calculated from the highest temperature measured in the experiment (43°C), and T_max_ cannot be predictably estimated and so is not shown*.

b *We further rejected any model predictions of maximum temperature where the uncertainty bounds (±SD) in the thermal optima overlapped with the uncertainty bounds in maximum temperature (±SD), as these predictions of T_max_ are questionable*.

There was no consistent trend in the effect of latitude or season on the maximum rate of photosynthesis at *T*_opt_ (Maximum rate, Table 1). Maximum *P*_gross(AG)_ in *H. uninervis* did not vary substantially between seasons, (i.e., overlapping SD at Moreton Bay), and maximum *P*_gross(AG)_ was higher in Moreton Bay, which was the subtropical site, than rates at Green Island in the tropics. Maximum *P*_gross(AG)_ of *C. serrulata* was lower than *H. uninervis* and it did not vary among latitudes in summer, but was lower in winter. Maximum *P*_gross(AG)_ of *Z. muelleri* in Moreton Bay in summer was within the range of rates measured for the other species (Table 1).

The *T*_opt_ is the temperature at which the photosynthesis rate is at the maximum (Figure 2). Error estimates for most thermal optima were ~1°C, so *T*_opt_ values have been rounded to the nearest degree in the text in this paper. The *T*_opt_ were 34–36°C in *H. uninervis* and *C. serrulata* and 31°C in *Z. muelleri* (Table 1). *T*_opt_ of *H. uninervis* varied among season and latitude combinations by ~2°C, but the variation did not follow ambient water temperature, while *T*_opt_ of *P*_gross(AG)_ of *C. serrulata* showed less variation between sites and seasons (~1°C) (Table 1). At temperatures above *T*_opt_, there was a rapid decline in photosynthetic rate, which was the most pronounced in *C. serrulata* and the most gradual for *Z. muelleri* (Figure 3).

Dark respiration of leaves increased with temperature (Q_10_ = 1.5–2.6, Table S1.1) for most of the temperature range examined (e.g., at temperatures less than ~35°C for all species, latitudes and seasons), and this increase was typically slower than the Q_10_ of *P*_gross(AG)_ (Figure S1.2). Conversely, at higher temperatures, dark respiration declined in the leaves of *H. uninervis* in summer at both latitudes and in *C. serrulata* at Moreton Bay in both summer and winter (Figure 3). Since the thermal optima for dark respiration of leaves sometimes exceeded the highest temperature we measured (43°C), we did not further compare the maximum respiration rates between species, seasons, and latitudes; this is also true for dark respiration rate of below-ground tissues.

Dark respiration rate of the below-ground tissues (*R*_BG_) increased with temperature more slowly than in leaves (Q_10_ = 1.3–1.9 for *C. serrulata* and *H. uninervis*, and 2.6 ± 0.9 for *Z. muelleri*, Table S1). The maximum rate of respiration, *R*_*AG*_, was 1.8–2.7 times higher than *R*_*BG*_ in *H. uninervis*, up to two times faster for *C. serrulata* and 1.3 times faster for *Z. muelleri* (Figure 3, Figure S1.2). At the highest temperature (43°C), *R*_*BG*_ declined in the below-ground parts of all species in summer at Moreton Bay and leaves of *H. uninervis* in summer at both sites (Figure 3).

### Temperature dependence of net productivity at the leaf scale

**Figure 4:**
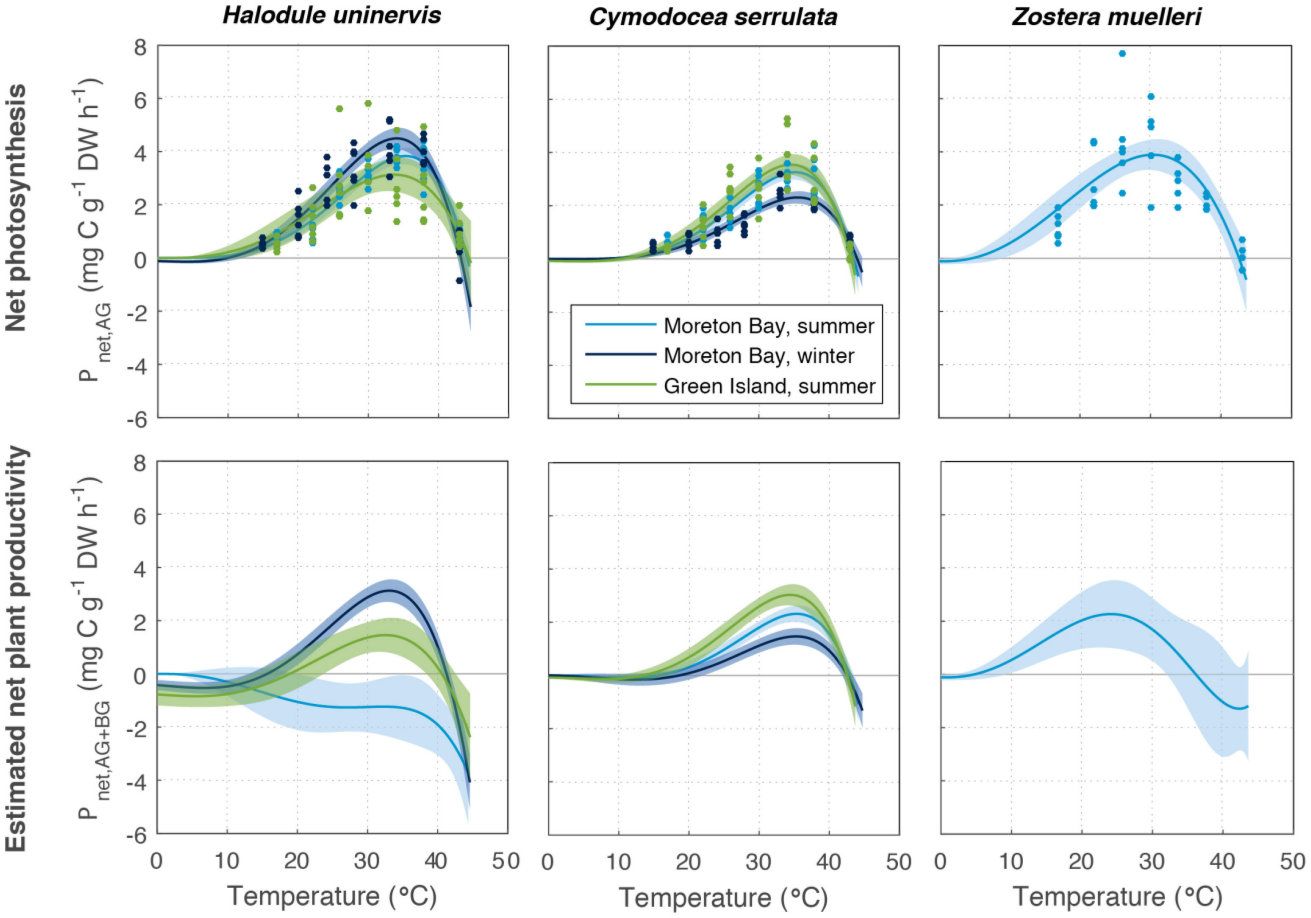
Net productivity of above-ground (*P*_*net*(*AG*)_, **Top**) and above and below-ground tissues together (*P*_*net*(*AG*+*BG*)_, based on mean below-ground to above-ground biomass ratio, **Bottom**) of *Halodule uninervis, Cymodocea serrulata*, and *Zostera muelleri*. These were measured at Green Island in summer and Moreton Bay in summer and winter. Ribbons are 95% CIs.

Net photosynthesis of leaves (*P*_net(AG)_), which accounted for leaf respiratory carbon loss, was in surplus from 15 to 43°C in most species and season-latitude combinations except for some replicates at 43°C (Figure 4). *P*_net(AG)_ followed the same temperature-dependent pattern as *P*_gross(AG)_ (compare Figures 2, 3). However, because *P*_net(AG)_ accounts for respiratory carbon loss, maximum *P*_net(AG)_ was 17% lower, on average, than that of *P*_gross(AG)_ in all species, and in both seasons and latitudes (Table S1.2). In contrast, *T*_opt_ of *P*_net(AG)_ was not substantially different to *T*_opt_ of *P*_gross(AG)_ (Table S2, Figure S1.3), indicating that the temperature-response dynamics of seagrass photosynthesis were largely unchanged after accounting for leaf respiration.

### Temperature dependence of net plant productivity

Estimated net plant productivity *P*_net(AG+BG)_ was calculated using Equation (4) from *P*_gross(AG)_ and respiratory carbon loss in above-ground (*R*_AG_) and below-ground tissues (*R*_BG_), and is shown for the mean allocation to below-ground and above-ground biomass in Figure 4. *P*_net(AG+BG)_ had a lower maximum rate, reaching 1.5–3.1 mg C g^−1^ DW h^−1^, among all species, and was 34% lower than *P*_net(AG)_ on average among all season and latitude combinations (Table S1.2). For the mean below-ground to above-ground biomass ratios measured, *P*_net(AG+BG)_ typically followed a similar temperature-dependent pattern as *P*_gross(AG)_ and *P*_net(AG)_ (Figures 3, 4). The exception was *P*_net(AG+BG)_ of *H. uninervis* in Moreton Bay in summer whereby *P*_net(AG+BG)_ declined with temperature (no initial rise) as the respiratory carbon loss from below-ground respiration was very large. Unlike *P*_gross(AG)_ and *P*_net(AG)_, a deficit in *P*_net(AG+BG)_ (<0) was estimated for all species, seasons and latitudes at temperatures less than 43°C. However, temperatures associated with net deficit in *P*_net(AG+BG)_ were variable among species, and sites/sampling times (Figure 4).

The below-ground to above-ground biomass ratio was, on average, 2.3–3.9 for *C. serrulata*, 5.0–10.6 for *H. uninervis*, and 9.6 for *Z. muelleri* (Table S1.3). There was large variability in this ratio among seasons, due largely to changes in above-ground biomass and among replicate samples: *C*. *serrulata* ranged from 0.8 to 12.1, *H. uninervis* ranged from 2.3 to 15.2, and *Z. muelleri* ranged from 2.6 to 20.4.

**Figure 5:**
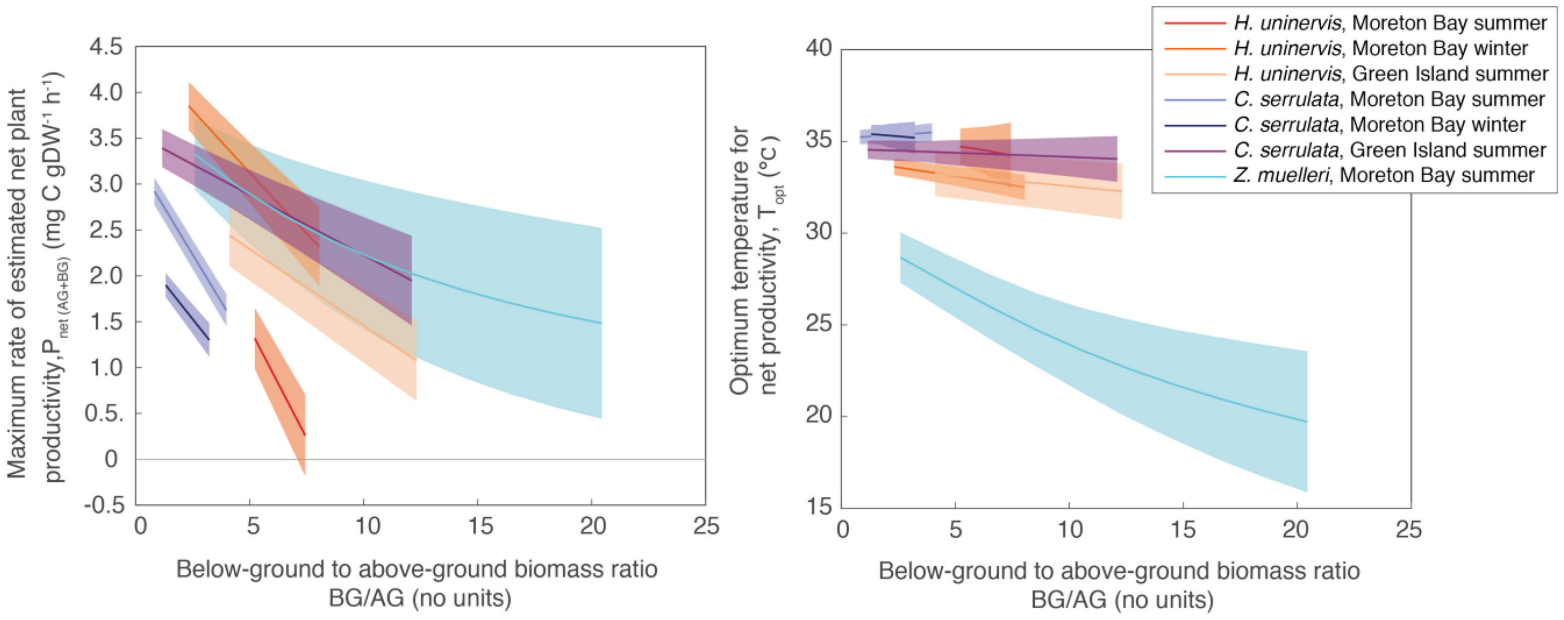
*P*_*max*_ **(Left)** and *T*_opt_ **(Right)** calculated from the relationship between *P*_net(AG+BG)_ and temperature using a range of below-ground to above-ground biomass ratios measured *in situ* (Table S3). Ribbons are 95% CIs.

The relative allocation of biomass to either below-ground or above-ground biomass had a large effect on the calculated *P*_net(AG+BG)_ in all species and a large effect on *T*_opt_ in two species (Table S1.2). At Green Island in summer, an increase in the below-ground to above-ground biomass ratio from 1.2 to 12.1 caused the predicted maximum *P*_net(AG+BG)_ in *C. serrulata* to drop by 45% and in *H. uninervis*, an increase in the ratio from 4.1 to 12.3 caused the predicted maximum *P*_net(AG+BG)_ to drop by 55% (Table S1.2, Figure 5). *Z. muelleri* had the widest range of *in situ* biomass ratios; when the ratio increased from 2.6 to 20.4 there was a decline in predicted maximum *P*_net(AG+BG)_ by 55%. There was negligible difference in the *T*_opt_ of *C. serrulata* for *P*_net(AG+BG)_ compared to the corresponding *T*_opt_ of *P*_gross(AG)_, remaining at ~35°C (Table S1.2, Figure 5). There was, however, a small reduction in *T*_opt_ for productivity of *H. uninervis* when accounting for respiration, declining from 34 to 36°C for *P*_gross(AG)_ to 33°C for *P*_net(AG+BG)_.

**Figure 6:**
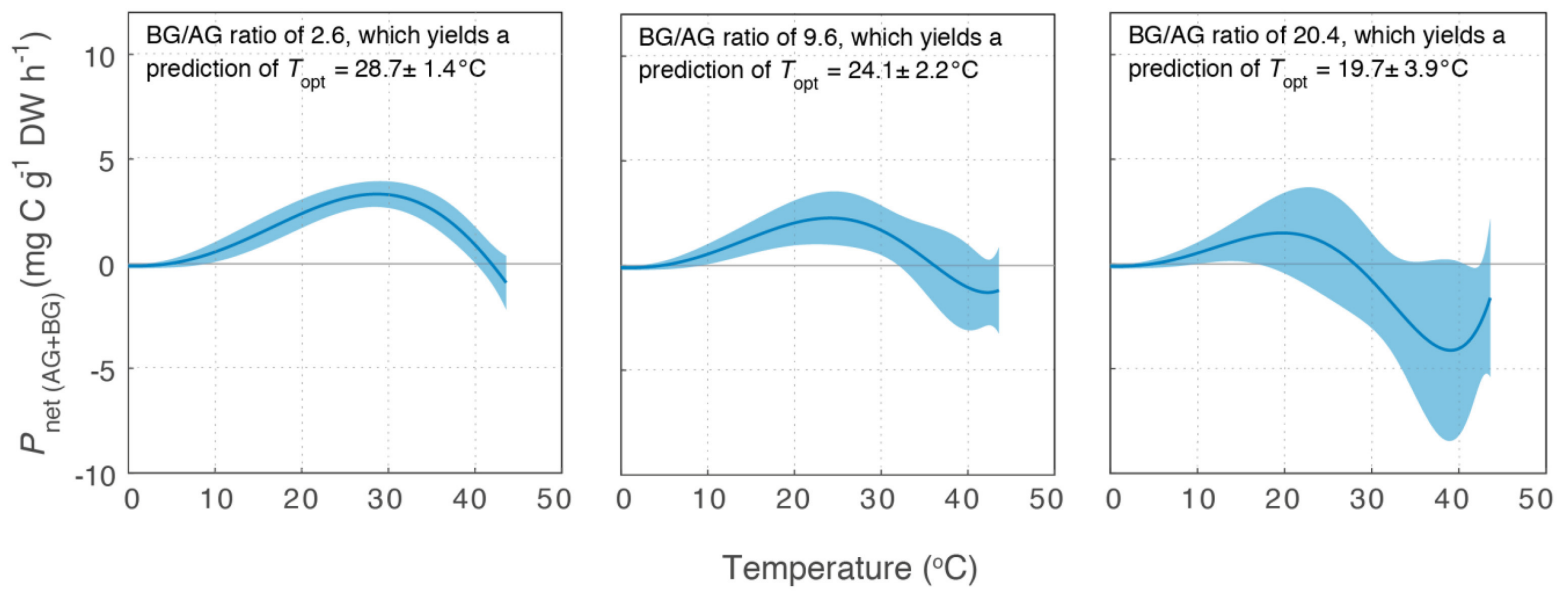
Modeled net plant productivity of the above-ground and below-ground tissues together (*P*_net(AG+BG)_) vs. temperature for *Z. muelleri*. *P*_net(AG+BG)_ was calculated from *P*_gross(AG)_, *R*_AG_, and *R*_BG_ for three different below-ground to above-ground biomass ratios: the minimum **(Left)**, mean **(Middle)**, and maximum **(Right)** below-ground to above-ground biomass ratio measured for *Z. muelleri* at the Moreton Bay site in summer. Ribbons are ±SD.

Variability in below-ground to above-ground biomass ratio had the greatest impact on the thermal optimum of *Z. muelleri*, compared to the other two species: *T*_opt_ of *P*_net(AG+BG)_ for *Z. muelleri* was 2°C less than *T*_opt_ of *P*_gross(AG)_ at the lowest below-ground to above-ground biomass ratio (1.6), but was 11°C less than *T*_opt_ of *P*_gross(AG)_ at the highest below-ground to above-ground biomass ratio (20.4). The thermal optimum for net plant productivity of *Z. muelleri* was predicted to range from 20 to 29°C, depending on the below-ground to above-ground biomass ratio. Based on the mean below-ground to above-ground biomass ratio (9.6), the thermal optimum for net plant productivity of *Z. muelleri* was 24°C (Figure 6).

## Discussion

*Z. muelleri* in Moreton Bay is already growing in conditions that exceed its thermal optimum and future warming will cause declines in net productivity. *C. serrulata* and *H. uninervis* have higher thermal optima that did not exceed ambient water temperature at the study sites; however, they inhabit northern Australia and the Indo-Pacific, including the Coral Triangle (Waycott et al., 2004), where water temperature (e.g., in February 2016, http://www.ospo.noaa.gov/Products/ocean/sst/50km_night/index.html) can approach the thermal optima of *H. uninervis* and *C. serrulata* for months, and also exceed thermal optima over tidal cycles as outlined further below. These thermal optima of net plant productivity were calculated from temperature-productivity responses of leaf photosynthesis, leaf respiration, and below-ground respiration and each of these processes has a maximum efficiency at a particular optimum temperature, *T*_opt_ (Table 1). The thermal optimum of net plant productivity (*P*_net(AG+BG)_) is an important threshold for plant survival, and, because it can be well below the thermal optimum for gross photosynthesis, the distinction between these two thresholds is critical for accurately predicting thermal stress.

### Thermal optima of photosynthesis

The thermal optima for photosynthesis (*P*_gross(AG)_) of *H. uninervis* and *C. serrulata*, whose distributions are restricted to the tropics and subtropics, were higher than that of *Z. muelleri*, a sub-tropical to temperate species (i.e., typically inhabiting cooler water). This suggests *adaptation* of each species to water temperature within its distributional range. The thermal optima (*T*_opt_) of photosynthesis (*P*_gross(AG)_) were 35°C for *H. uninervis* and *C. serrulata*, averaged over the latitude and season combinations and 31°C for *Z. muelleri*. However, there was limited within-species *acclimation* (and/or adaptation across sites) of thermal optima based on ambient water temperature (latitude and season) in *H. uninervis* and *C. serrulata*.

In contrast, thermal optima for photosystem II efficiency (Δ*F*/*F*_m_') were less sensitive to changes in water temperature; Δ*F*/*F*_m_' did not decline in *H. uninervis* and *C. serrulata* when temperature was increased from ambient (26°C) to 35 and 40°C (Campbell et al., 2006; Collier and Waycott, 2014). The lower optima for *P*_gross(AG)_ in this study based on oxygen evolution compared to Δ*F*/*F*_m_', is likely due to the sensitivity of Rubisco to thermal stress (Salvucci and Crafts-Brandner, 2004). Beyond the thermal optima, photosynthetic rates declined rapidly after *T*_opt_, reaching o = 0 mg C g^−1^ DW h^−1^ at *T*_max_ of 44–45°C in all species.

The optimum temperature for photosynthesis varied little over the latitude and season combinations (1°C for *C. serrulata* and 2°C for *H. uninervis*). Furthermore, the variation did not follow ambient water temperature as *T*_opt_ was higher in winter than in summer and higher in Moreton Bay than at Green Island for *H. uninervis* and Topt did vary among locations in *C. serrulata*. Optimum temperature for photosynthesis was well above ambient water temperature at the time of measurement (21.0–28.8°C), and above long-term ambient daily water temperature across latitudes in all species (15.3–30.8°C). Thermal acclimation over seasonally varying water temperatures enables *Z. marina* from the northern hemisphere to maintain energetic surplus over large changes in water temperature (Zimmerman et al., 1989; Staehr and Borum, 2011). *Z. marina* is a temperate species that grows at temperatures ranging from sub-zero to 32°C and has thermal optima for maximum photosynthesis ranging from 16 to 24°C to maintain productivity under highly variable water temperatures (Lee et al., 2007). Water temperature in tropical regions has lower annual variability than in sub-tropical and temperate regions and the species *H. uninervis* and *C. serrulata* by contrast do not appear to have developed the physiological flexibility to acclimate to changing water temperature. Similarly, tropical rainforest trees have higher optimum temperature compared to related temperate species in some genera, but more importantly, tropical species maintain maximum photosynthetic rates over narrower temperature ranges because the ambient temperature range is also lower (Read, 1990; Cunningham and Read, 2002).

### Respiration and net productivity of leaves

Net leaf productivity (*P*_net(AG)_) accounts for leaf respiratory carbon loss and was 17% lower on average than *P*_gross(AG)_. Leaf respiration rates increased with temperature (Q_10_ = 1.6–1.9, except in the leaves of *H. uninervis* from Moreton Bay in summer for which Q_10_ was 2.64), and these increased respiration rates can be driven by higher rates of biomass production at higher water temperatures (van der Heide et al., 2006), as well as repair and maintenance mechanisms, such as the production of stress proteins that enable optimization of metabolic processes under wide-ranging temperatures (Koutalianou et al., 2015).

Thermal optima for net leaf productivity were slightly lower (0.5°C on average) than thermal optima for leaf photosynthesis. In *H. uninervis*, thermal optima for net leaf productivity was 34–35°C (mean 34°C) and in *C. serrulata* it was 35°C on average. These thermal optima, derived from empirical curve-fitting, are higher than thermal optima for net productivity of leaves from predominantly sub-tropical meadows as summarized in Lee et al. (2007) (23–32°C). Thermal optima in this study were also slightly higher than previously reported values of *T*_opt_ of leaves for the tropical species *E. acroides* (33.3°C) and *T. hemprichii* (32.1°C) (Pedersen et al., 2016), which are species that can co-occur with *H. uninervis* and *C. serrulata* in tropical meadows.

### Net plant productivity

Net plant productivity accounts for respiratory carbon loss from below-ground tissues. Below-ground respiration rates (in o = mg C g^−1^ DW h^−1^) were considerably lower (2–5 times lower) than above-ground respiration rates; however, the differences in respiration rates between above- and below-ground tissues were smaller than for *T. testudinum* (Fourqurean and Zieman, 1991) and *Z. marina* (Zimmerman et al., 1989; Staehr and Borum, 2011), which both found respiration of the leaves was seven times higher than for rhizomes. Therefore, the respiratory burden of the below-ground tissues was lower in the species we examined, but due to the large proportion of below-ground biomass, net productivity (mg C g DW^−1^ h^−1^) of the whole plant (*P*_net(AG+BG)_) was 34% lower than net productivity of leaves only (based on mean biomass ratio among all species and treatment combinations). This highlights the importance of accounting for below-ground respiration in seagrass productivity estimates (Zimmerman et al., 1989; Fourqurean and Zieman, 1991).

Estimated net plant productivity was affected by the allocation to below-ground and above-ground biomass in all species such that when the proportion of below-ground biomass was low, net plant productivity was highest. This verifies the hypothesis developed in *Z. muelleri* net community metabolisms (NCM) studies where shallow (high light) meadows had NCM around zero, while deeper (low light) meadows had positive NCM; the lower NCM in shallow meadows was attributed to their higher below ground biomass rather than differences in gross productivity associated with light levels (Ferguson et al., 2017). The present study also demonstrates that thermal optima are affected by the below-ground to above-ground biomass ratios. The three species examined in this study are considered opportunistic, with high morphological plasticity (Kilminster et al., 2015; Ferguson et al., 2016). The mean biomass ratio for *Z. muelleri* was higher in this study (9.6) than the mean previously reported which ranged from 0.9 to 5 (McKenzie, 1994; Duarte and Chiscano, 1999; Ferguson et al., 2017), while the mean ratios for *H. uninervis* (5.0–10.6) and *C. serrulata* (2.3–3.9) are consistent with previously reported values (5.6 and 1.1–4.4, respectively) (de Boer, 2000; Collier et al., 2012; Gokulakrishnan and and Ravikumar, 2016). Biomass allocation can be affected by nutrient and sediment quality gradients (Udy et al., 1999; Ferguson et al., 2016), light gradients (Collier et al., 2007) and may also have been affected by exposure to low tide and waves at the Moreton Bay site as shorter leaves (low leaf biomass) tend to form in exposed meadows (Koch, 2001). This study has shown that site-specific biomass allocation strategies must be considered when calculating thermal optima of net plant productivity for other meadows.

Net plant productivity is not frequently reported due the difficulty of measuring and scaling productivity of clonal plants with varying biomass allocation. Productivity estimates for the source meadow can be calculated from *P*_net(AG+BG)_ (see Supplementary Section S1.1 for meadow productivity estimates), and can be calculated for other meadows, assuming no thermal acclimation, using site-specific biomass ratios (See Supplement 2 for methods). These meadow-scale productivity estimates should not be confused with community metabolism studies, which also measure productivity of interstitial and epiphytic organisms (e.g., Duarte et al., 2010; Ferguson et al., 2017).

There are a number of potential applications for *T*_opt_ in assessing seagrass vulnerability to ocean warming. *T*_opt_ defines the temperature above which net plant productivity declines: comparing *T*_opt_ with ambient temperature will reveal whether ocean warming will increase or reduce net plant productivity. Despite limited seasonal acclimation, thermal optima of net plant productivity for *C. serrulata* (35°C) and *H. uninervis* (33°C) were higher than mean daily water temperature at the study sites (15.3°C–30.8°C). Therefore, net productivity is unlikely to reach optimal rates in the field for these two species but this higher physiological optimum ensures that heat stress is largely avoided. Furthermore, a predicted future rise in sea surface temperature of 2°C by 2100 (RCP 4.5 in surface waters; IPCC, 2013) is unlikely to lead to chronic exceedance of thermal optima at these sites, and instead, it will increase net productivity. For example, using the temperature production model developed from measured data in this study, net plant productivity of *C. serrulata* will increase by 25% in Moreton Bay and by 11% at Green Island following a 2°C rise in maximum summer water temperature (i.e., a rise in maximum water temperature from 29.0 to 31°C and from 30.8 to 32.8°C at Moreton Bay and Green Island, respectively). Using the spreadsheet in Supplement 2, the change in net plant productivity can be estimated for any given rise in water temperature. It is important to note, however, that rises in water temperature could increase vulnerability to other stressors including light limitation (Collier et al., 2016), contaminants (Wilkinson et al., 2017), disease susceptibility (Kaldy, 2014), and sulfide intrusion (Koch et al., 2007; Kilminster et al., 2008).

The limited acclimation to ambient water temperature suggests that the thermal optima of *H. uninervis* and *C. serrulata* may be extrapolated to other regions. Ambient water temperature already does reach thermal optima of *H. uninervis* and *C. serrulata* throughout the distributional range in the Indo-Pacific (e.g., in February 2016, http://www.ospo.noaa.gov/Products/ocean/sst/50km_night/index.html) and therefore, these species when present in other regions near the edge of their range are likely to be at risk from thermal stress which will be exacerbated in the future. Pooling in shallow habitats due to receding tides could also expose seagrasses to high temperatures; for example at a different site on Green Island, water temperature in shallow water pools at low tide exceeded 33°C for short periods (hours) on 13% of days and exceeded 35°C on 3% of days from 2004 to 2015 (McKenzie et al., 2016). Therefore, both increases in sea surface temperature combined with tidally induced exposure to extremes in edge-of-range and shallow meadows could place these species at risk of thermal stress (Massa et al., 2009; Pedersen et al., 2016). The next step is to assess cumulative temperature stress (analogous to degree heating weeks) (Eakin et al., 2009), by correlating deviation from *T*_opt_ with meadow condition.

Ambient water temperature in Moreton Bay frequently exceeds the thermal optima for *P*_net(AG+BG)_ (24°C based on mean below- to above-ground biomass ratio) of *Z. muelleri* highlighting that these meadows are already at some risk of thermal stress. Therefore, a 2°C rise in ambient water temperature in Moreton Bay will lead to declining plant net productivity. Using the temperature-production model for this species in Moreton Bay, we can calculate that a 2°C rise in water temperature will lead to a 21% reduction in net plant productivity. However, net plant productivity declined gradually above *T*_opt_ in *Z. muelleri* (compared to *C. serrulata* and *H. uninervis*) and it remained net productive at temperatures up to 35°C, indicating a broad thermal window for survival.

The thermal optimum for plant productivity may not necessarily reflect the optimum growth temperature due to the temperature sensitivity of roots to anoxia and thermal disruption of carbohydrate metabolism and transport (Zimmerman et al., 1989). The thermal optima for net productivity based on below- to above-ground biomass found in this study appear to be consistent with previous findings on long-term survival under different growth temperatures. *Z. muelleri*, had highest shoot survival rates at 24 to 27°C when grown at 24 to 33°C for 3 months (York et al., 2013), and at 27°C when grown at 27 to 33°C for 1 month (Collier et al., 2011), which is within the range of the *T*_opt_ for net plant productivity (20–29°C, Figure 5). Similarly, after 1 month at temperatures ranging from 27 to 33°C, *H. uninervis* had highest net productivity and leaf growth rates at 33°C, which is also the *T*_opt_ for net plant productivity for this species (Figure 6). Therefore, *T*_opt_ for estimated net plant productivity provides a proxy for optimal growth temperatures in these species. Temperature sensitivity, thermal optima and acclimatory potential can vary with phenological stage, and therefore seedling development and sexual reproduction may have different thermal optima to that of mature plants (Atkin and Tjoelker, 2003; Wahid et al., 2007).

Respiration rates of above- and below-ground tissues typically increased with temperature up to 43°C, but in almost all cases, there was an optimum temperature for respiration (*T*_opt_) where rates were highest prior to a downturn at the highest temperatures. In other samples, the respiration continued to increase over the measured temperatures. Therefore, *T*_opt_ of both leaf respiration and below-ground respiration had a wide range (from 34°C to over 43°C). At elevated temperatures, enzymes deactivate, membranes become instable, secondary metabolites, and heat shock proteins are produced and the production of reactive oxygen species increases (Wahid et al., 2007; Bita and Gerats, 2013), and at extreme temperature, respiratory function is impaired and respiration rates plummet (Atkin and Tjoelker, 2003; O'Sullivan et al., 2013), but this process of declining respiration at extreme temperatures has not been observed in tropical seagrasses before, even at much higher temperatures of up to 50°C (Pedersen et al., 2016).

In conclusion, this study has identified the thermal optima for net plant productivity of three tropical seagrass species. These temperature thresholds can be compared to local water temperatures, to assess the risk ocean warming poses to seagrass meadows. The thermal optima for photosynthesis were highest in the tropical-distributed species *H. uninervis* and *C. serrulata*, and lowest in the sub-tropical to temperate *Z. muelleri* with limited acclimation among latitude and seasons. Thermal optima were lower for net productivity, particularly for plant net productivity that also includes respiratory carbon loss from below-ground tissue in *Z. muelleri*. With larger proportion of below-ground biomass, estimated net productivity and *T*_opt_ were further reduced. Therefore, the *T*_opt_ values we report for net plant productivity may be applied as a temperature threshold immediately to other ecosystems containing the same seagrass species, in conjunction with site-specific measurements of below-ground to above-ground biomass ratio.

## Author contributions

CC, YO, LL, SU, and MA conceived the experiments. CC, YO, LL, SU, VH, and CJ conducted the experiments. LL, YO, and CC prepared the data. MA, CC, and KO analyzed the results. CC, MA, YO, LL, SU, CJ, KO, and VH prepared the manuscript.

### Conflict of interest statement

The authors declare that the research was conducted in the absence of any commercial or financial relationships that could be construed as a potential conflict of interest.
