# Supplementary material for: Optimum Temperatures for Net Primary Productivity of Three Tropical Seagrass Species
Source: Front Plant Sci. 2017 Aug 23;8:1446. doi: 10.3389/fpls.2017.01446 (PMC5572403; doi:10.3389/fpls.2017.01446)
Supplement: Supplementary file 1 [file Table-1.docx]

# Supplementary material 1

Collier, C. J., Y. X. Ow, L. Langlois, S. Uthicke, C. Johansson, K. O'Brien, V. Hrebien, and M. P. Adams. 2017. Primary productivity and thermal optima of three tropical seagrass species. Frontiers in Plant Science 8:1446.

Figure S1.1. Species composition shown by allocation to total biomass (n = 8 ± SE of total combined biomass).

Figure S1.2. The factor increase in photosynthesis (*P*_net(AG)_) and respiration (*R*_AG_ and *R*_BG_) rates over 10°C, also known as Q_10_, at temperatures below the thermal optima for *H. uninervis*, *C. serrulata* and *Z. muelleri* at Moreton Bay (MB) and Green Island (GI) in summer and winter. n = 6 ± SD

Figure S1.3. *T*_opt_ of photosynthesis (*P*_gross(AG)_) and productivity (*P*_net(AG)_) for *H. uninervis*, *C. serrulata* and *Z. muelleri* at Moreton Bay and Green Island in summer and winter. n = 6 ± SD

Table S1.1. Estimates of photosynthetic and respiratory rates at 20ºC, and Q_10_ values, for leaves and below-ground biomass of tropical seagrass in Queensland, Australia. n = 6 ± SD

| **Species** | **Location, Season** | **Rate at 20ºC (mg C g^-1^ DW h^-1^)** | | |  | **Q_10_ (no units)** | | |
| --- | --- | --- | --- | --- | --- | --- | --- | --- |
|  |  | ***P*_gross(AG)_** | ***R*_AG_** | ***R*_BG_** |  | ***P*_gross(AG)_** | ***R*_AG_** | ***R*_BG_** |
| *H. uninervis* | Moreton Bay, Summer | 1.6±0.12 | 0.33±0.04 | 0.20±0.04 |  | 2.38±0.15 | 2.64±0.28 | 1.89±0.28 |
|  | Moreton Bay, Winter | 2.05±0.19 | 0.40±0.04 | 0.15±0.02 |  | 2.10±0.19 | 1.86±0.11 | 1.56±0.12 |
|  | Green Island, Summer | 1.51±0.33 | 0.31±0.04 | 0.12±0.01 |  | 2.72±0.72 | 1.87±0.25 | 1.26±0.09 |
| *C. serrulata* | Moreton Bay, Summer | 1.31±0.11 | 0.30±0.02 | 0.21±0.03 |  | 2.22±0.16 | 1.62±0.08 | 1.63±0.18 |
|  | Moreton Bay, Winter | 0.82±0.07 | 0.22±0.02 | 0.17±0.03 |  | 2.70±0.22 | 1.78±0.12 | 1.63±0.24 |
|  | Green Island, Summer | 1.32±0.18 | 0.20±0.02 | 0.07±0.01 |  | 2.35±0.28 | 1.61±0.11 | 1.63±0.23 |
| *Z. muelleri* | Moreton Bay, Summer | 2.50±0.37 | 0.27±0.06 | 0.08±0.03 |  | 2.21±0.42 | 1.58±0.21 | 2.60±0.88 |

Table S1.2. Predicted *T*_opt_ and rate of maximum productivity at *T*_opt_ for gross photosynthesis (*P*_gross(AG)_), net productivity of the leaves (*P*_net(AG)_ = *P*_gross(AG)_ – *R*_AG_), and estimated net productivity of the plant (*P*_net(AG+BG)_ = *P*_gross(AG)_ – *R*_AG_ – BG/AG × *R*_BG_). *P*_net(AG+BG)_ is calculated three times: for the minimum, mean and maximum below-ground to above-ground (BG/AG) biomass ratios. The BG/AG biomass ratios are calculated from biomass data specific to the species, season and location. T_opt_ and the rate of maximum productivity may decrease when (1) leaf and below-ground respiration are taken into account and (2) BG/AG biomass ratio increases. ^a^Predicted *T*_opt_ falls outside the temperature range measured in this experiment, so is inaccurate and thus not shown. ^b^This BG/AG biomass ratio data includes measurements made at a nearby site on the north-western side of Green Island. n = 6 ± SD

| **Species** | **Location, Season** | **Productivity measure** | **Maximum rate,**  **at *T_opt_* (mg C g^-1^ DW h^-1^)** | ***T_opt_* (ºC)** |
| --- | --- | --- | --- | --- |
| *H. uninervis* | Moreton Bay, Summer | *P*_gross(AG)_ | 5.2±0.1 | 35.8±0.2 |
|  |  | *P*_net(AG)_ | 3.8±0.3 | 35.3±0.4 |
|  |  | *P*_net(AG+BG)_, min. BG:AG ratio of 5.2 | 1.3±0.7 | 34.7±1.0 |
|  |  | *P*_net(AG+BG)_, mean BG:AG ratio of 10.6^a^ | - | - |
|  |  | *P*_net(AG+BG)_, max. BG:AG ratio of 15.2^a^ | - | - |
|  | Moreton Bay, Winter | *P*_gross(AG)_ | 5.5±0.2 | 34.9±0.3 |
|  |  | *P*_net(AG)_ | 4.5±0.4 | 34.0±0.4 |
|  |  | *P*_net(AG+BG)_, min. BG:AG ratio of 2.3 | 3.9±0.4 | 33.6±0.4 |
|  |  | *P*_net(AG+BG)_, mean BG:AG ratio of 5.0 | 3.1±0.4 | 33.1±0.5 |
|  |  | *P*_net(AG+BG)_, max. BG:AG ratio of 8.1 | 2.3±0.5 | 32.5±0.7 |
|  | Green Island, Summer | *P*_gross(AG)_ | 3.9±0.3 | 34.0±0.9 |
|  |  | *P*_net(AG)_ | 3.1±0.6 | 33.7±1.1 |
|  |  | *P*_net(AG+BG)_, min. BG:AG ratio of 4.1 | 2.4±0.6 | 33.3±1.2 |
|  |  | *P*_net(AG+BG)_, mean BG:AG ratio of 10.0 | 1.5±0.6 | 32.6±1.4 |
|  |  | *P*_net(AG+BG)_, max. BG:AG ratio of 12.3 | 1.1±0.7 | 32.3±1.5 |
| *C. serrulata* | Moreton Bay, Summer | *P*_gross(AG)_ | 3.9±0.1 | 35.4±0.3 |
|  |  | *P*_net(AG)_ | 3.3±0.3 | 35.2±0.4 |
|  |  | *P*_net(AG+BG)_, min. BG:AG ratio of 0.8 | 2.9±0.3 | 35.2±0.4 |
|  |  | *P*_net(AG+BG)_, mean BG:AG ratio of 2.3 | 2.3±0.3 | 35.3±0.4 |
|  |  | *P*_net(AG+BG)_, max. BG:AG ratio of 4.0 | 1.6±0.4 | 35.5±0.5 |
|  | Moreton Bay, Winter | *P*_gross(AG)_ | 2.9±0.1 | 35.8±0.3 |
|  |  | *P*_net(AG)_ | 2.3±0.2 | 35.5±0.4 |
|  |  | *P*_net(AG+BG)_, min. BG:AG ratio of 1.3 | 1.9±0.3 | 35.4±0.5 |
|  |  | *P*_net(AG+BG)_, mean BG:AG ratio of 2.7 | 1.5±0.3 | 35.3±0.8 |
|  |  | *P*_net(AG+BG)_, max. BG:AG ratio of 3.2 | 1.3±0.3 | 35.2±0.9 |
|  | Green Island, Summer | *P*_gross(AG)_ | 4.0±0.2 | 34.9±0.5 |
|  |  | *P*_net(AG)_ | 3.6±0.4 | 34.6±0.5 |
|  |  | *P*_net(AG+BG)_, min. BG:AG ratio of 1.2^b^ | 3.4±0.4 | 34.6±0.5 |
|  |  | *P*_net(AG+BG)_, mean BG:AG ratio of 3.9^b^ | 3.0±0.4 | 34.4±0.6 |
|  |  | *P*_net(AG+BG)_, max. BG:AG ratio of 12.1^b^ | 1.9±0.5 | 34.1±1.3 |
| *Z. muelleri* | Moreton Bay, Summer | *P*_gross(AG)_ | 4.3±0.3 | 30.9±1.0 |
|  |  | *P*_net(AG)_ | 3.9±0.6 | 30.4±1.1 |
|  |  | *P*_net(AG+BG)_, min. BG:AG ratio of 2.6 | 3.3±0.6 | 28.7±1.4 |
|  |  | *P*_net(AG+BG)_, mean BG:AG ratio of 9.6 | 2.3±1.3 | 24.1±2.2 |
|  |  | *P*_net(AG+BG)_, max. BG:AG ratio of 20.4 | 1.5±1.9 | 19.7±3.9 |

Table S1.3. Below- to above-ground biomass ratio (BG/AG) in up to eight replicate samples for the species for which temperature response curves were measured from the study site and the mean. Data only shown if the species was found in the random sample (n = 8 ± SE).

|  | **Summer, Green Island** | | **Summer, Moreton Bay** | | | **Winter, Moreton Bay** | |
| --- | --- | --- | --- | --- | --- | --- | --- |
| **Replicates** | ***H. uninervis*** | ***C. serrulata^a^*** | ***H. uninervis*** | ***C. serrulata*** | ***Z. muelleri*** | ***H. uninervis*** | ***C. serrulata*** |
| **1** | 10.9 | 3.9 | 5.2 | 1.7 | 5.8 | 4.4 | 1.3 |
| 2 | 10.8 | 1.2 | 13.9 | 0.8 | 20.4 | 5.5 | 3.0 |
| 3 | 10.7 | 1.9 | 9.8 | 2.2 | 2.6 | 5.5 | 3.1 |
| 4 | 4.1 | 1.6 | 7.7 | 3.0 |  | 3.1 | 2.5 |
| 5 | 10.6 | 3.3 | 15.2 | 2.0 |  | 6.0 | 3.2 |
| 6 | 12.3 | 3.6 | 9.7 | 4.0 |  | 8.1 | 3.0 |
| 7 | 9.9 | 3.6 | 12.7 | 3.3 |  | 2.3 |  |
| 8 | 10.8 | 12.1 |  | 1.4 |  |  |  |
| **Average** | **10.0 (4.7)** | **3.9 (3.8)** | **10.6 (3.5)** | **2.3 (1.0)** | **9.6 (9.5)** | **5.0 (1.9)** | **2.3 (1.2)** |

^a^Biomass ratio data from a nearby site (~500m away) used for biomass ratios as there was insufficient at the study site.

***Supplementary Section S1.1: Productivity at ambient temperature***

Light-saturated productivity at ambient water temperature was calculated from the temperature-production response curves. This was then converted to areal net productivity based on *in situ* biomass. *P*_net(AG)_ at ambient water temperature ranged from 0.67 to 3.72 mgC gDW^-1^ h^-1^ (Table S4). *P*_net(AG)_ was 50% and 217% higher in summer for *H. uninervis* and *C. serrulata* respectively. *P*_net(AG)_ was comparable between locations for *H. uninervis* but 30% higher at Green Island than at Moreton Bay for *C. serrulata*. *Z. muelleri* had the highest *P*_net(AG)_. Areal above-ground productivity was more than five times greater in summer than in winter at Moreton Bay, and in summer it was almost twice as high than in summer at Green Island due to the larger biomass. Total productivity at ambient water temperature (*P*_net(AG+BG)_) ranged from 0.61 to 2.38 mgC gDW^-1^ h^-1^, except for *H. uninervis* at Moreton Bay which had a negative productivity due to high below-ground respiratory loads (Table 2). This resulted in a total light-saturated net productivity of the meadow (all species combined) being 46.48 to 204.24 mgC m^-2^ h^-1^ at ambient water temperature.

Table S1.4. *In situ* total biomass at the study sites and also predicted light-saturated productivity of above-ground biomass only (leaf-scale *P*_net(AG)_ in mg C gDW^-1^ h^-1^ and meadow scale, *P*_net(AG)_ in mgC m^-2^ h^-1^) and of above- and below-ground biomass combined (leaf-scale *P*_net(AG+BG)_ in mg C gDW^-1^ h^-1^ and meadow scale, *P*_net(AG+BG)_ in mgC m^-2^ h^-1^) at ambient temperature at the study sites and areal productivity. Biomass n = 8 ± SE, productivity n = 6 ± SD.

|  |  |  | **Above-ground productivity** | | **Total productivity** | |
| --- | --- | --- | --- | --- | --- | --- |
| **Location** | **Species** | **Biomass (gDW m^-2^)** | ***P*_net(AG)_**  **(mgC g^-1^ DW h^-1^)**  **at ambient temp** | ***P*_net(AG)_**  **(gC m^-2^ h^-1^)** | ***P*_net(AG+BG)_**  **(mgC g^-1^DW h^-1^)**  **at ambient temp** | ***P*_net(AG+BG)_**  **(mgC m^-2^ h^-1^)** |
| Moreton Bay, summer  Ambient Temp = 27.2ºC | *H. uninervis* | 139.7 (40.4) | 2.6 (0.2) | 32.9 | -1.3 (0.7) | -176.0 |
|  | *C. serrulata* | 118.3 (72.1) | 2.1 (0.2) | 80.0 | 1.3 (0.2) | 157.3 |
|  | *Z. muelleri* | 38.7 (14.9) | 3.7 (0.3) | 11.1 | 2.1 (0.9) | 81.7 |
|  | **Total** | **256.7 (26.8)** | **2.8^*^** | **127.5^*^** | **0.7^*^** | **66.2** |
| Moreton Bay, winter  Ambient Temp = 21.0ºC | *H. uninervis* | 34.5 (7.1) | 1.7 (0.2) | 6.2 | 0.9 (0.2) | 32.4 |
|  | *C. serrulata* | 27.1 (11.5) | 0.7 (0.1) | 7.4 | 0.2 (0.4) | 4.3 |
|  | **Total** | **77.5 (15.9)** | **1.7^*^** | **22.7^*^** | **0.6^*^** | **46.5** |
| Green Island, summer Ambient Temp = 29.5ºC | *H. uninervis* | 119.1 (18.5) | 2.9 (0.3) | 31.6 | 1.3 (0.4) | 158.4 |
|  | *C. serrulata* | 18.3 (0) | 3.0 (0.2) | 11.2 | 2.5 (0.3) | 45.8 |
|  | **Total** | **154.2 (23.7)** | **2.4^*^** | **71.4^*^** | **1.4^*^** | **204.2** |

*mean *P*_net_ used for other species present at the site, but not included in the temperature-production measures.
